# Supplementary material for: A Functional ClpXP Protease is Required for Induction of the Accessory Toxin Genes, tst, sed, and sec
Source: Toxins (Basel). 2020 Aug 28;12(9):553. doi: 10.3390/toxins12090553 (PMC7551677; doi:10.3390/toxins12090553)
Supplement: Supplementary file 1 [file toxins-12-00553-s001.pdf]

Supplementary Materials: A Functional ClpXP Protease is Required for Induction of the Accessory Toxin Genes, *tst, sed,* and *sec*

Jenny Schelin, Marianne Thorup Cohn, Babro Frisk. Dorte Frees

Table S1. Significantly differentially expressed* genes between RN4282 wild-type and RN4282 expressing the ClpXI265E variant.

Pan Gene id [A]	Pan Gene Symbol [B]	Fold Change[C]	p_{adj}	Product
**SAUPAN006313000**	betA	**32.5**	6.70×10^{-55}	oxygen-dependent choline dehydrogenase
**SAUPAN006314000**	betB	**31.8**	1.79×10^{-73}	betaine–aldehyde dehydrogenase
**SAUPAN001141000**	lrgA	**11.9**	2.27×10^{-12}	antiholin-like protein LrgA
**SAUPAN006063000**	SAUSA300_2423	**10.9**	3.49×10^{-2}	hypothetical protein
**SAUPAN005063000**	SAUSA300_1937	**8.5**	1.34×10^{-3}	Sa3int prophage protein
**SAUPAN001142000**	lrgB	**8.3**	1.75×10^{-16}	antiholin-like protein LrgB
**SAUPAN005056000**	NWMN_1891	**7.1**	9.11×10^{-6}	Sa3int prophage protein
**SAUPAN005055000**	SAUSA300_1934	**6.4**	1.17×10^{-8}	Sa3int prophage encoded protein(tail protein)
**SAUPAN000943000**	sasD	**5.8**	3.49×10^{-6}	LPXTG cell wall anchor domain
**SAUPAN006110000**	**sarU**	**5.8**	5.91×10^{-14}	Transcriptional regulator
**SAUPAN005244000**	ear	**5.7**	1.94×10^{-7}	**SaPI1 encoded protin Ear,**
**SAUPAN005623000**	SAUSA300_2164	**5.4**	2.86×10^{-7}	Conserved protein. Signal peptide.
**SAUPAN005361000**	SAUSA300_2041	**5.0**	2.72×10^{-10}	hypothetical protein
**NWMN_RS10890**	NWMN_RS10890	**5.0**	3.30×10^{-5}	Hypothetical Sa3int prophage protein
**SAUPAN005899000**	SAUSA300_2325	**5.0**	6.27×10^{-14}	putative type II DNA modification enzyme
**JKDJBECI_01742**	-	**5.0**	2.77×10^{-9}	tRNA-Arg(ccg)
**SAUPAN005138000**	SAUSA300_1938	**4.9**	4.82×10^{-6}	Sa3int prophage protein. putative capsid protein
**SAUPAN001245000**	gehB	**4.8**	6.17×10^{-18}	lipase
**SAUPAN006318000**	cudT	**4.6**	3.10×10^{-11}	choline transporter BetT
**SAUPAN002480000**	SAUSA300_0602	**4.5**	7.57×10^{-12}	hypothetical protein
**SAUPAN005928000**	narG	**4.4**	4.32×10^{-3}	nitrate reductase subunit alpha
**SAUPAN005933000**	nirR	**4.4**	1.45×10^{-2}	nitrite reductase transcriptional regulator NirR
**SAUPAN002548000**	**sarX** (SAUSA300_0654)	**4.1**	4.06×10^{-13}	MarR family transcriptional regulator
**SAUPAN005061000**	SAUSA300_1936	**4.1**	4.15×10^{-2}	conserved hypothetical phage protein
**SAUSA300_RS10655**	-	**4.0**	3.45×10^{-2}	hypothetical protein
**SAUSA300_RS05270**	-	**3.9**	1.06×10^{-5}	hypothetical protein
**SAUPAN005046000**	SAUSA300_1929	**3.9**	1.59×10^{-3}	Sa3int prophage encoded protein (tail component)
**JKDJBECI_02004**	-	**3.9**	8.40×10^{-3}	tRNA-Ile(gat)
**SAUPAN001175000**	esxA	**3.7**	1.21×10^{-8}	T7SS EsxA

**SAUPAN002816000**	ohr (SAUSA300_0786)	**3.7**	1.94 × 10⁻³	Putative organic hydroperoxide reductase
**SAUPAN005052000**	SAUSA300_1932	**3.7**	2.21 × 10⁻²	Sa3int prophage encoded protein
**SAUSA300_RS10625**	-	**3.7**	1.99 × 10⁻²	Sa3int prophage encoded protein
**SAUPAN001863000**	ulaA	**3.7**	4.55 × 10⁻⁸	PTS ascorbate transporter subunit IIC.
**SAUPAN006030000**	cntK	**3.6**	4.87 × 10⁻⁵	Histidine isomerase
**SAUSA300_RS10500**	-	**3.5**	3.17 × 10⁻⁴	MAP domain-containing protein
**SAUPAN003448000**	mraZ	**3.5**	7.81 × 10⁻⁹	division/cell wall cluster transcriptional repressor
**SAUPAN003164000**	**spxA**	**3.5**	9.11 × 10⁻¹⁴	regulatory protein Spx
**SAUPAN002481000**	SAUSA300_0603	**3.5**	5.24 × 10⁻⁶	hypothetical membrane protein
**SAUPAN000662000**	SAUSA300_1941	**3.4**	5.56 × 10⁻³	Sa3int prophage encoded protein (terminase)
**SAUPAN003449000**	mraW	**3.3**	1.46 × 10⁻⁵	Putative S-adenosyl-methyltransferase
**SAUPAN005389000**	SAUSA300_2056	**3.3**	2.53 × 10⁻⁴	conserved membrane protein
**SAUPAN005245000**	tst	**3.2**	1.13 × 10⁻³	**toxic shock syndrome toxin-1 TSST-1**
**SAUPAN002802000**	cspC	**3.2**	3.82 × 10⁻³	cold-shock protein
**SAUPAN003211000**	**comK1**	**3.2**	1.27 × 10⁻⁶	ComK competence transcription factor
**SAUPAN005139000**	SAUSA300_1939	**3.2**	9.72 × 10⁻⁵	Sa3int prophage encoded protein
**SAUPAN000910000**	**sarS**	**3.1**	2.14 × 10⁻⁷	Virulence regulator
**SAUPAN004572000**	epiG	**3.1**	9.94 × 10⁻⁸	antibiotic epidermin immunity protein F,
**SAUPAN004573000**	epiE	**3.0**	9.35 × 10⁻⁷	lantibiotic epidermin immunity protein F.
**SAUPAN004574000**	epiF	**3.0**	1.41 × 10⁻⁴	bacitracin ABC transporter ATP-binding protein
**SAUPAN004575000**	epiP	**3.0**	3.24 × 10⁻⁵	lantibiotic epidermin. protease EpiP
**SAUPAN004939000**	SAUSA300_1890	**3.0**	1.31 × 10⁻⁴	Cysteine protease. staphopain
**SAUPAN004396000**	fhs	**3.0**	1.27 × 10⁻⁶	formate—tetrahydrofolate ligase
**SAUPAN004585000**	epiA	**3.0**	5.98 × 10⁻⁵	lantibiotic epidermin
**SAUPAN003450000**	ftsL	**3.0**	1.42 × 10⁻⁴	cell division protein FtsL
**SAUPAN001228000**	SAUSA300_0307	**3.0**	8.15 × 10⁻⁶	5'-nucleotidase. lipoprotein e(P4) family
**SAUSA300_RS10495**	-	**2.9**	3.04 × 10⁻³	MAP domain-containing protein
**SAUPAN004024000**	**srrB**	**2.9**	2.67 × 10⁻⁵	two-component sensor histidine kinase
**JKDJBECI_00383**	-	**2.9**	1.94 × 10⁻⁷	hypothetical protein
**SAUPAN003637000**	SAUSA300_1205	**2.9**	3.77 × 10⁻²	hypothetical protein
**SAUPAN002374000**	SAUSA300_0575	**2.9**	2.07 × 10⁻⁷	hypothetical protein
**SAUPAN005018000**	scn	**2.8**	6.20 × 10⁻⁴	staphylococcal complement inhibitor SCIN
**SAUPAN002347000**	hxlA	**2.8**	1.15 × 10⁻⁴	putative hexulose−6-phosphate synthase.

**SAUSA300_RS13715**	-	**2.8**	1.10×10^{-5}	hypothetical membrane protein
**SAUPAN006035000**	SAUSA300_2416	**2.8**	3.08×10^{-6}	glucose 1-dehydrogenase–like protein
**SAUPAN002348000**	hxlB	**2.8**	3.51×10^{-5}	6-phospho-3-hexuloisomerase.
**SAUPAN002823000**	SAUSA300_0793	**2.8**	1.10×10^{-4}	conserved protein.
**SAUPAN001865000**	SAUSA300_0332	**2.8**	3.31×10^{-3}	Putative PTS transporter.
**SAUPAN004576000**	epiD	**2.6**	3.48×10^{-2}	lantibiotic epidermin biosynthesis protein EpiD
**SAUPAN005023000**	sak	**2.6**	1.17×10^{-3}	Sa3int prophage encoded staphylokinase
**SAUPAN001971000**	SAUSA300_0372	**2.6**	1.36×10^{-2}	conserved protein
**SAUPAN004358000**	SAUSA300_1653	**2.6**	2.58×10^{-3}	metal-dependent hydrolase
**SAUSA300_RS10645**	-	**2.5**	3.77×10^{-3}	Sa3int prophage encoded protein
**SAUPAN003698000**	thrD	**2.5**	8.22×10^{-5}	aspartate kinase
**SAUPAN006490000**	rnpA	**2.5**	3.67×10^{-4}	ribonuclease P protein component
**SAUPAN006028000**	SAUSA300_2412	**2.5**	5.06×10^{-3}	conserved protein.
**SAUSA300_RS14095**	-	**2.5**	3.55×10^{-5}	hypothetical protein
**SAUPAN005951000**	SAUSA300_2361	**2.5**	1.08×10^{-5}	hypothetical protein
**SAUPAN001864000**	SAUSA300_0331	**2.5**	5.06×10^{-3}	Putative PTS transporter.
**SAUPAN004025000**	**srrA**	**2.5**	1.28×10^{-4}	DNA-binding response regulator
**SAUPAN006464000**	SAUSA300_2632	**2.5**	1.31×10^{-3}	Conserved membrane protein
**SAUPAN003129000**	-	**2.5**	3.78×10^{-4}	Membrane protein
**SAUPAN005949000**	mdeA/SAUSA300_2360	**2.4**	3.08×10^{-6}	Putative efflux pump
**SAUPAN000942000**	sodM	**2.4**	1.32×10^{-3}	Superoxide dismutase [Mn/Fe] 2
**SAUPAN001005000**	SAUSA300_0171	**2.4**	1.93×10^{-3}	putative cation transporter
**SAUPAN006029000**	cntL	**2.4**	3.09×10^{-2}	Staphylopine biosynthesis
**SAUPAN002813000**	SAUSA300_0785	**2.4**	1.54×10^{-2}	N-acetyltransferase
**SAUPAN006197000**	cidB	**2.4**	4.94×10^{-4}	holin-like protein CidB
**SAUSA300_RS10345**	-	**2.4**	1.63×10^{-2}	staphostatin A
**SAUPAN003631000**	SAUSA300_1203	**2.4**	1.93×10^{-2}	hypothetical protein
**SAUPAN005239000**	SAUSA300_1980	**2.4**	1.89×10^{-3}	N-acetyltransferase
**JKDJBECI_00385**	-	**2.4**	2.62×10^{-5}	hypothetical protein
**SAUPAN003126000**	SAUSA300_0884	**2.4**	6.42×10^{-3}	hypothetical protein
**SAUPAN005281000**	**agrA**	**2.3**	3.71×10^{-6}	DNA-binding response regulator
**SAUPAN002892000**	SAUSA300_0816	**2.3**	1.34×10^{-3}	Conserved protein of unknown function
**SAUPAN005048000**	-	**2.3**	2.36×10^{-3}	Sa3int prophage protein
**SAUPAN006033000**	SAUSA300_2415	**2.3**	1.26×10^{-3}	hypothetical protein
**SAUPAN002822000**	SAUSA300_0792	**2.3**	1.36×10^{-2}	hypothetical protein
**JKDJBECI_00384**	-	**2.3**	1.14×10^{-4}	hypothetical protein
**SAUPAN005192000**	lukH	**2.3**	4.28×10^{-2}	succinyl-diaminopimelate desuccinylase
**SAUPAN000037000**	adsA	**2.3**	1.30×10^{-5}	AdsA nucleotidase
**SAUPAN005279000**	**agrC**	**2.3**	8.37×10^{-6}	Qurum sensing
**SAUPAN003133000**	opp-3B	**2.3**	3.67×10^{-4}	ABC transporter permease
**SAUPAN005496000**	mtlR/SAUSA300_2106	**2.3**	9.14×10^{-3}	conseved protein
**SAUPAN002835000**	SAUSA300_0794	**2.3**	1.09×10^{-2}	topiosmerase.
**SAUPAN005897000**	scrA	**2.3**	3.54×10^{-3}	PTS sucrose transporter subunit IIBC

**SAUPAN005275000**	**agrB**	**2.3**	1.56×10^{-5}	accessory gene regulator AgrB
**SAUPAN005498000**	mtlA	**2.3**	5.88×10^{-4}	mannitol-specific phosphotransferase
**SAUSA300_RS10880**	-	**2.2**	9.14×10^{-3}	hypothetical protein
**SAUPAN001008000**	SAUSA300_0172	**2.2**	3.51×10^{-2}	conserved hypothetical protein
**SAUPAN003654000**	SAUSA300_1210	**2.2**	3.97×10^{-3}	hypothetical protein
**SAUPAN005883000**	SAUSA300_2315	**2.2**	1.35×10^{-2}	Conserved secreted protein
**SAUPAN001034000**	murQ	**2.2**	3.31×10^{-3}	N-acetylmuramic acid 6-phosphate etherase
**SAUPAN005388000**	murA	**2.2**	1.49×10^{-3}	peptidoglycan biosynthesis
**SAUPAN002836000**	SAUSA300_0795	**2.2**	3.35×10^{-2}	Putative thiol reductase thioredoxin.
**SAUPAN002669000**	SAUSA300_0739	**2.2**	1.89×10^{-3}	Putative autolysin (CHAP and LysM domain)
**SAUSA300_RS03975**	prf2	**2.2**	1.28×10^{-4}	peptide chain release factor 2
**SAUPAN005915000**	narK	**2.2**	1.43×10^{-2}	nitrite extrusion protein
**SAUPAN005580000**	amaP/SAUSA300_2144	**2.2**	4.70×10^{-3}	hypothetical protein
**SAUPAN000894000**	norC	**2.2**	8.58×10^{-3}	Efflux pump NorC
**SAUPAN006196000**	cidC	**2.1**	3.25×10^{-4}	pyruvate oxidase
**SAUPAN006107000**	sasG	**2.1**	1.56×10^{-4}	LPXTG-motif cell wall anchor
**SAUSA300_RS09325**	-	**2.1**	3.10×10^{-4}	MarR family transcriptional regulator
**SAUPAN003251000**	sspA	**2.1**	2.73×10^{-4}	V8 serine protease.
**SAUPAN004030000**	xerD	**2.1**	6.65×10^{-4}	tyrosine recombinase XerD
**SAUPAN001012000**	ssuC/SAUSA300_0176	**2.1**	1.08×10^{-1}	ABC transporter permease.
**SAUPAN005440000**	manA	**2.1**	1.28×10^{-4}	mannose–6-phosphate isomerase.
**SAUPAN003692000**	SAUSA300_1221	**2.1**	1.19×10^{-2}	conserved protein
**SAUPAN004317000**	phoP	**2.1**	1.58×10^{-3}	DNA-binding response regulator
**SAUPAN001862000**	SAUSA300_0329	**2.1**	1.67×10^{-2}	putative oxidoreductase
**SAUPAN004046000**	SAUSA300_1458	**2.1**	1.93×10^{-3}	conserved protein
**SAUPAN002570000**	**mgrA**	**2.1**	3.17×10^{-4}	MarR family transcriptional regulator
**SAUPAN003936000**	engA	**2.1**	2.70×10^{-4}	ribosome–associated GTPase EngA
**SAUPAN003057000**	SAUSA300_0858	**2.1**	1.34×10^{-4}	conserved protein
**SAUPAN003134000**	oppC	**2.1**	8.97×10^{-3}	oligopeptide ABC transporter permease
**SAUPAN001123000**	tarI/SAUSA300_0245	**2.1**	5.71×10^{-4}	TarI
**SAUPAN003451000**	pbpA	**2.1**	2.29×10^{-2}	penicillin-binding protein 1
**SAUPAN003135000**	oppD	**2.1**	1.31×10^{-2}	ABC transporter ATP-binding protein.
**SAUPAN005276000**	**agrD**	**2.1**	1.36×10^{-2}	cyclic lactone autoinducer peptide
**SAUPAN005500000**	mtlD	**2.0**	3.00×10^{-3}	mannitol-1-phosphate 5-dehydrogenase
**SAUPAN005274000**	**Hld/RNAIII**	**2.0**	8.80×10^{-3}	delta-hemolysin/RNAIII
**SAUPAN005578000**	asp23	**2.0**	4.94×10^{-3}	Asp23 envelope stress response protein
**SAUPAN005387000**	fabZ	**2.0**	7.46×10^{-3}	fatty acid biosynthesis
**SAUPAN006489000**	trmE	**2.0**	1.13×10^{-3}	tRNA modification GTPase TrmE
**SAUPAN002232000**	SAUSA300_0471/veg	**2.0**	9.89×10^{-3}	hypothetical protein

**SAUPAN003935000**	gpsA	**2.0**	5.22 × 10^{-4}	glycerol-3-phosphate dehydrogenase (NAD(P)(+))
**SAUPAN003803000**	SAUSA300_1284/cvfB	**2.0**	3.15 × 10^{-3}	S1 RNA binding domain-containing protein
**SAUPAN005439000**	SAUSA300_2095	**2.0**	9.03 × 10^{-4}	conserved protein
**SAUPAN001900000**	metE	**2.0**	1.89 × 10^{-3}	homocysteine methyltransferase
**SAUPAN002714000**	rnr	**2.0**	3.76 × 10^{-3}	ribonuclease R
**SAUPAN001013000**	SAUSA300_0177	**2.0**	4.00 × 10^{-2}	conserved protein.
**SAUPAN003808000**	dapA	**2.0**	3.62 × 10^{-2}	4-hydroxy-tetrahydrodipicolinate synthase
**SAUPAN004316000**	phoR	**2.0**	4.41 × 10^{-3}	sensor histidine kinase
**JKDJBECI_00373**	-	**2.0**	6.47 × 10^{-3}	hypothetical protein
**SAUPAN003249000**	sspC	**2.0**	1.70 × 10^{-3}	staphostatin B
**SAUPAN006210000**	clpL	**2.0**	1.88 × 10^{-2}	ATP-dependent chaperone ClpL
**SAUPAN005803000**	glvC	**2.0**	1.32 × 10^{-3}	PTS alpha-glucoside transporter subunit IIBC
**SAUPAN003746000**	glcT	**0.5**	3.77 × 10^{-3}	protein GlcT
**SAUPAN002314000**	rpoB	**0.5**	3.77 × 10^{-3}	DNA-directed RNA polymerase subunit beta
**SAUPAN006189000**	-	**0.5**	2.97 × 10^{-2}	alpha/beta hydrolase
**SAUPAN006326000**	cysJ	**0.5**	1.56 × 10^{-2}	(NADPH) flavoprotein subunit
**SAUPAN001981000**	ahpF	**0.5**	1.51 × 10^{-2}	alkyl hydroperoxide reductase subunit F
**SAUPAN002328000**	-	**0.5**	1.93 × 10^{-2}	HAD family hydrolase
**SAUPAN003440000**	psmβ1	**0.5**	3.96 × 10^{-2}	PSM β1
**SAUPAN000975000**	capB	**0.5**	3.35 × 10^{-2}	capsular polysaccharide biosynthesis protein Cap5B
**SAUPAN003392000**	-	**0.5**	2.60 × 10^{-2}	metal-dependent phosphodiesterase
**SAUPAN005723000**	-	**0.5**	2.63 × 10^{-2}	MFS transporter
**SAUPAN004195000**	-	**0.5**	1.15 × 10^{-2}	hypothetical protein
**SAUPAN006293000**	panB	**0.5**	1.24 × 10^{-2}	hydroxymethyltransferase
**SAUPAN005704000**	-	**0.5**	5.46 × 10^{-3}	hypothetical protein
**SAUPAN000035000**	walI	**0.5**	8.12 × 10^{-3}	hypothetical protein
**SAUPAN006202000**	-	**0.5**	1.41 × 10^{-2}	CHAP domain-containing protein
**SAUPAN005444000**	czrB	**0.5**	3.45 × 10^{-2}	cation transporter
**SAUPAN002315000**	rpoC	**0.5**	1.32 × 10^{-3}	DNA-directed RNA polymerase subunit beta'
**SAUPAN005301000**	tsaE	**0.5**	7.48 × 10^{-3}	tRNA (adenosine(37)-N6)-TsaE
**SAUPAN000935000**	galE	**0.5**	3.75 × 10^{-2}	NAD-dependent dehydratase
**SAUPAN002502000**	-	**0.5**	8.94 × 10^{-3}	sodium:proton antiporter
**SAUPAN002164000**	mpsB	**0.5**	3.87 × 10^{-3}	hypothetical protein
**SAUPAN003241000**	-	**0.5**	1.44 × 10^{-2}	acetyltransferase
**SAUPAN006136000**	-	**0.5**	1.41 × 10^{-2}	hypothetical protein
**SAUPAN006109000**	**sarT**	**0.5**	1.57 × 10^{-2}	transcriptional regulator
**SAUPAN005268000**	sdrH	**0.5**	7.94 × 10^{-3}	hypothetical protein
**SAUPAN005300000**	tsaB	**0.5**	1.37 × 10^{-2}	tRNA threonylcarbamoyltransferase c
**SAUPAN006292000**	panC	**0.5**	4.94 × 10^{-3}	pantoate—beta-alanine ligase

**SAUPAN001869000**	mepA	**0.5**	4.55×10^{-2}	multidrug efflux MATE transporter MepA
**SAUPAN002252000**	ftsH	**0.5**	3.86×10^{-4}	zinc metalloprotease
**SAUPAN004943000**	-	**0.5**	3.58×10^{-2}	NETI motif-containing protein
**SAUPAN001098000**	prsS	**0.5**	8.31×10^{-3}	PrsW family intramembrane metalloprotease
**SAUPAN006448000**	-	**0.5**	1.93×10^{-2}	hypothetical protein
**SAUPAN006325000**	-	**0.5**	2.02×10^{-2}	precorrin-2 dehydrogenase
**SAUPAN001173000**	-	**0.5**	2.60×10^{-2}	DUF5080 domain-containing protein
**SAUPAN002321000**	-	**0.5**	2.66×10^{-2}	N-acetyl-L,L-diaminopimelate deacetylase
**SAUPAN005993000**	-	**0.5**	2.63×10^{-3}	NAD(P)-dependent oxidoreductase
**SAUPAN003787000**	opp-2B	**0.5**	1.94×10^{-3}	peptide ABC transporter permease
**SAUPAN001984000**	nfrA	**0.5**	8.31×10^{-3}	NADPH-dependent oxidoreductase
**SAUPAN003331000**	mntH	**0.5**	7.37×10^{-3}	divalent metal cation transporter
**SAUPAN002533000**	-	**0.5**	1.41×10^{-2}	alpha/beta hydrolase
**SAUPAN000387000**	-	**0.5**	1.70×10^{-3}	LysR family transcriptional regulator
**SAUPAN002101000**	-	**0.5**	2.24×10^{-3}	membrane protein
**SAUPAN002183000**	-	**0.5**	3.23×10^{-2}	hypothetical protein
**SAUPAN006233000**	-	**0.5**	3.79×10^{-2}	N-acetyltransferase
**SAUPAN004501000**	metK	**0.5**	3.00×10^{-3}	S-adenosylmethionine synthase
**SAUPAN006457000**	rarD	**0.5**	7.64×10^{-4}	protein RarD
**SAUPAN005501000**	fmtB	**0.5**	7.48×10^{-3}	hypothetical protein
**SAUPAN003171000**	pepF	**0.5**	2.37×10^{-3}	oligoendopeptidase F
**SAUPAN004277000**	tag	**0.5**	1.13×10^{-3}	DNA-3-methyladenine glycosylase I
**SAUPAN003441000**	psmβ2	**0.5**	1.31×10^{-3}	hemolytic protein
**SAUPAN005722000**	-	**0.5**	3.43×10^{-2}	MarR family transcriptional regulator
**SAUPAN002102000**	-	**0.5**	4.61×10^{-3}	NAD(P)-dependent oxidoreductase
**SAUPAN001102000**	opp-5A	**0.5**	5.22×10^{-4}	nickel ABC transporter substrate–binding protein
**SAUPAN006159000**	fbp	**0.5**	8.53×10^{-3}	fructose 1,6-bisphosphatase
**SAUPAN000148000**	-	**0.4**	3.18×10^{-3}	hypothetical protein
**SAUPAN003257000**	-	**0.4**	4.62×10^{-4}	acyltransferase
**SAUPAN005997000**	opuCD	**0.4**	6.78×10^{-4}	amino acid ABC transporter permease
**SAUPAN000938000**	-	**0.4**	3.31×10^{-3}	transferase
**SAUPAN005998000**	opuCC	**0.4**	4.44×10^{-4}	amino acid transporter
**SAUPAN005608000**	-	**0.4**	7.79×10^{-3}	MerR family transcriptional regulator
**SAUPAN006191000**	-	**0.4**	1.93×10^{-3}	acyl-CoA thioester hydrolase
**SAUPAN005606000**	-	**0.4**	1.56×10^{-3}	aldo/keto reductase
**SAUPAN001171000**	-	**0.4**	3.43×10^{-2}	DUF5079 domain-containing protein
**SAUPAN003058000**	-	**0.4**	1.32×10^{-3}	NADH-dependent flavin oxidoreductase

**SAUPAN000902000**	-	**0.4**	1.93×10^{-2}	oleate hydratase
**SAUPAN002142000**	lpl3	**0.4**	4.70×10^{-2}	tandem-type lipoprotein
**SAUPAN005375000**	thiD	**0.4**	3.77×10^{-3}	kinase/phosphomethylpyrimi dine kinase
**SAUPAN002631000**	nrdF	**0.4**	3.58×10^{-3}	ribonucleotide−diphosphate reductase subunit beta
**SAUPAN006437000**	-	**0.4**	3.95×10^{-6}	polysaccharide deacetylase
**SAUPAN000902000**	-	**0.4**	7.88×10^{-3}	oleate hydratase
**SAUPAN003593000**	-	**0.4**	1.26×10^{-3}	2-oxoacid ferredoxin oxidoreductase subunit beta
**SAUPAN002282000**	-	**0.4**	8.80×10^{-4}	PLP-dependent aminotransferase family protein
**SAUPAN006177000**	-	**0.4**	3.24×10^{-5}	peptide ABC transporter permease
**SAUPAN005741000**	fdhD	**0.4**	1.80×10^{-4}	sulfurtransferase FdhD
**SAUPAN003757000**	-	**0.4**	3.21×10^{-5}	DNA repair protein MucB
**SAUPAN006190000**	-	**0.4**	9.72×10^{-5}	thiol reductase thioredoxin
**SAUPAN006420000**	gehA	**0.4**	2.75×10^{-3}	Lipase 1
**SAUPAN005767000**	ssaA	**0.4**	4.51×10^{-6}	CHAP domain-containing protein
**SAUPAN005934000**	-	**0.4**	1.25×10^{-4}	N-acetyltransferase
**SAUPAN001163000**	lytM	**0.4**	7.59×10^{-4}	glycyl-glycine endopeptidase LytM
**SAUPAN003592000**	-	**0.4**	7.10×10^{-4}	2-oxoglutarate ferredoxin oxidoreductase
**SAUPAN003680000**	-	**0.4**	2.69×10^{-5}	low specificity L-threonine aldolase
**SAUPAN003201000**	-	**0.3**	4.55×10^{-9}	membrane protein
**SAUPAN006322000**	nrdD	**0.3**	1.98×10^{-2}	anaerobic ribonucleoside−triphosphate reductase
**SAUPAN005999000**	opuCB	**0.3**	2.71×10^{-3}	choline ABC transporter permease
**SAUPAN006408000**	cap1B	**0.3**	2.87×10^{-3}	capsular polysaccharide biosynthesis protein Cap5B
**SAUPAN003815000**	msa	**0.3**	5.43×10^{-6}	protein msa
**SAUPAN006409000**	cap1A	**0.3**	3.70×10^{-2}	capsular polysaccharide biosynthesis protein CapA
**SAUPAN006176000**	-	**0.3**	8.40×10^{-4}	peptide ABC transporter ATP-binding protein
**SAUPAN006459000**	nixA	**0.3**	1.27×10^{-6}	nickel transporter NixA
**SAUPAN005381000**	sceD	**0.3**	8.80×10^{-4}	transglycosylase SceD
**SAUPAN006218000**	-	**0.3**	2.16×10^{-5}	maltose O-acetyltransferase
**SAUPAN006000000**	opuCA	**0.3**	1.21×10^{-8}	glycine/betaine ABC transporter ATP-binding protein
**SAUPAN006366000**	argR	**0.3**	1.55×10^{-5}	ArgR family transcriptional regulator
**SAUSA300_RS06715**	-	**0.2**	1.10×10^{-5}	hypothetical protein
**SAUPAN006321000**	nrdG	**0.2**	1.62×10^{-5}	anaerobic ribonucleoside−triphosphate reductase activating
**NWMN_RS11100**	-	**0.2**	1.04×10^{-16}	hypothetical protein
**SAUPAN005772000**	-	**0.2**	1.18×10^{-6}	CHAP domain-containing protein

**SAUPAN005759000**	ureD	**0.2**	9.35×10^{-26}	urease accessory protein
**SAUPAN005756000**	ureE	**0.2**	8.85×10^{-23}	urease accessory protein UreE
**SAUPAN005758000**	ureG	**0.2**	2.88×10^{-23}	urease accessory protein UreG
**SAUPAN005757000**	ureF	**0.2**	2.23×10^{-26}	urease accessory protein UreF
**SAUPAN005755000**	ureC	**0.2**	1.72×10^{-21}	urease subunit alpha
**SAUPAN005753000**	ureA	**0.2**	3.77×10^{-16}	urease subunit gamma
**SAUPAN005754000**	ureB	**0.2**	3.52×10^{-21}	urease subunit beta
**SAUPAN005752000**	-	**0.1**	3.08×10^{-18}	urea transporter
**NWMN_RS10980**	-	**0.0**	6.24×10^{-27}	hypothetical protein
**SAUPAN005109000**	-	**0.0**	4.93×10^{-26}	hypothetical protein

* Genes were considered differentially expressed if they showed a ≥2-fold change in expression with $p_{adj} < 0.05$. [A]: RN8242 genes were annotated using the AureoWiki database (http://aureowiki.med.uni-greifswald.de) using preferentially the pan-genome gene id and otherwise with identifiers corresponding to the strain-specific gene to which they map. Open reading frames in the RN4282 genome that could not be assigned to any of the five strains in Aureowiki (NCTC 8325, COL. Newman, USA300_FPR3757, and N315) were annotated using a prokka-generated identifier and reported as hypothetical proteins. [B]: Genes encoding known transcriptional regulators of virulence genes are shown in bold. In case no pan genome symbol was provided in AureoWiki, the gene annotations for the S. aureus model strain USA300_FPR3757 were used. [C]: Fold changes in RN4282 wild type/RN4282clpXI265E are shown (genes expressed >2 fold more in wild-type cells are listed in the top of the table (shaded pink). Genes expressed >2 fold more in RN4282clpXI265E cells are listed in the bottom of the table shaded green).
